# STMN1表达与非小细胞肺癌预后价值的*meta*分析

**DOI:** 10.3779/j.issn.1009-3419.2024.102.39

**Published:** 2024-11-20

**Authors:** Mengjie LI, Qinghua ZHOU

**Affiliations:** ^1^300060 天津，天津医科大学肿瘤医院肺部肿瘤内科，国家恶性肿瘤临床医学研究中心，天津市恶性肿瘤临床医学研究中心，天津市肿瘤防治重点实验室（李梦洁）; ^1^Department of Thoracic Oncology, Tianjin Medical University Cancer Institute & Hospital; National Clinical Research Center for Cancer; Tianjin’s Clinical Research Center for Cancer; Key Laboratory of Cancer Prevention and Therapy, Tianjin 300060, China; ^2^610041 成都，四川大学华西医院肺癌中心，四川省肺癌研究所（周清华）; ^2^Lung Cancer Center, West China Hospital, Sichuan University; Sichuan Lung Cancer Institute, Chengdu 610041, China

**Keywords:** 肺肿瘤, STMN1, 预后, Meta分析, Lung neoplasms, STMN1, Prognosis, Meta-analysis

## Abstract

**背景与目的:**

肺癌是全球发病率和死亡率最高的恶性肿瘤之一，严重危害人类健康，其中非小细胞肺癌（non-small cell lung cancer, NSCLC）占所有肺癌病例的85%以上。STMN1是一种广泛存在于细胞质中的微管解聚蛋白，其STMN1表达水平与NSCLC患者预后相关。本研究旨在通过meta分析探究STMN1表达水平对于肺癌预后的预测价值，筛选高敏感高特异性的生物标志物以优化肺癌患者全程管理。

**方法:**

检索PubMed、The Cochrane Library、Embase、万方、知网等数据库建库至2024年9月6日发表的相关文献，采用纽卡斯尔-渥太华量表（Newcastle-Ottawa Scale, NOS）对文献质量进行评分。采用风险比（hazard ratio, HR）及对应的95%CI探究STMN1表达水平与NSCLC患者预后的相关性。预后指标包括总生存期（overall survival, OS）和无病生存期（disease-free survival, DFS）。所有统计分析由STATA 17.0软件完成。

**结果:**

共纳入5项高质量研究（NOS评分≥6分），包括754例研究对象。Meta分析结果显示，STMN1过表达与NSCLC患者OS（HR=2.28, 95%CI: 1.79-2.91, P<0.001）和DFS（HR=2.14, 95%CI: 1.45-3.17, P<0.001）较差明显相关，STMN1表达是NSCLC患者预后不良的危险因素。

**结论:**

STMN1过表达的NSCLC患者预后更差。STMN1表达水平可作为NSCLC患者的预后生物标志物，但上述结论仍需更多研究进一步论证。

## Abstract (en)

**Background and objective:**

Lung cancer is one of the malignant tumors with the highest morbidity and mortality rates worldwide, seriously threatening human health. Non-small cell lung cancer (NSCLC) accounts for more than 85% of all lung cancer cases. STMN1 is a microtubule depolymerizing protein widely present in the cytoplasm and its expression level is associated with the prognosis of NSCLC patients. Through meta-analysis, this study aimed to investigate the predictive value of the expression level of STMN1 for the prognosis of lung cancer and screen for tumor markers with high sensitivity and specificity to optimize the whole-process management of lung cancer patients.

**Methods:**

The PubMed, The Cochrane Library, Embase, WanFang and CNKI databases were searched from the inception to Sep 6, 2024 for relevant literature. The quality of included studies was assessed by the Newcastle-Ottawa Scale (NOS) score. The hazard ratio (HR) with 95%CI was combined to assess the relationship between STMN1 expression and prognostic factors. The prognostic indicators included the overall survival (OS) and disease-free survival (DFS). All statistical analysis was conducted by the STATA 17.0 software.

**Results:**

A total of 5 high-quality studies (NOS score≥6 points) involving 754 patients were enrolled. The pooled results demonstrated that overexpression of STMN1 was significantly related to worse OS (HR=2.28, 95%CI: 1.79-2.91, P<0.001) and DFS (HR=2.14, 95%CI: 1.45-3.17, P<0.001). Overexpression of STMN1 was a risk factor for poor prognosis of NSCLC patients.

**Conclusion:**

Overexpression of STMN1 is a poor prognostic factor in NSCLC patients. STMN1 may serve as a prognostic biomarker for NSCLC patients. However, more researches are still needed to verify the above findings.

肺癌是全球范围内导致癌症死亡的最主要原因，占所有癌症相关死亡的18%^[1]^。根据病理类型，肺癌分为非小细胞肺癌（non-small cell lung cancer, NSCLC）和小细胞肺癌，其中NSCLC占全部肺癌类型的85%以上^[2,3]^。近年来，随着低剂量螺旋计算机断层扫描（computed tomography, CT）的普及以及靶向治疗和免疫治疗的发展，NSCLC患者的5年生存率有所提高，但仍不理想^[4]^。转移是NSCLC患者预后不良的主要因素之一^[5]^，上皮间充质转化（epithelial-mesenchymal transition, EMT）被认为是癌症转移的关键过程^[6,7]^。探索潜在的治疗靶点以靶向抑制转移，对于改善肺癌患者预后至关重要。

STMN1是一种广泛存在于细胞质中的微管解聚蛋白^[8]^，其有四个丝氨酸磷酸化位点，去磷酸化状态是其活性形式，可以促进微管解聚^[9,10]^。STMN1在大多数肿瘤中呈高表达状态，例如乳腺癌^[11]^、胃癌^[12]^和肝细胞癌^[13]^。随着对STMN1的深入研究，发现它与多种生物学过程密切相关，如增殖^[14]^和耐药性^[15]^。回顾性临床研究^[16,17]^发现，在大多数肿瘤中，STMN1的表达与淋巴结转移和临床分期呈正相关。有研究^[18,19]^报道某些基因可通过抑制STMN1的磷酸化水平从而促进肿瘤转移。然而，也有关于STMN1在肿瘤转移中作用的不同观点，有研究^[20]^表明STMN1与EMT呈负相关。本研究通过meta分析探究STMN1的表达与NSCLC患者临床预后的相关性，以期为临床上NSCLC的预后评估和探索新的治疗靶点提供一定的循证医学证据。

## 1 资料与方法

### 1.1 数据收集

检索PubMed、The Cochrane Library、Embase、万方、知网等数据库中的相关文献，检索时间为建库到2024年9月6日。英文检索词包括：STMN1、Stathmin 1、lung、pulmonary、carcinoma、cancer、neoplasm、tumor、prognosis、survival；中文检索词包括：肺癌、肺恶性肿瘤、生存、预后。文献筛选过程见图1。

**图1:**
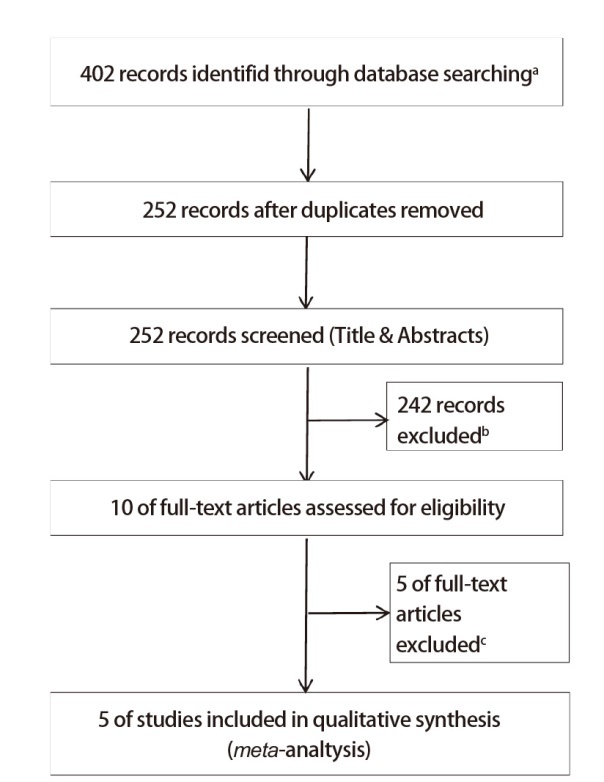
文献筛选流程及结果

### 1.2 纳入标准

（1）研究对象：年龄≥18岁，病理确诊NSCLC；（2）暴露因素或干预措施：根据患者接受治疗前STMN1表达水平分为高表达组和低表达组，进一步比较两组的预后差异；（3）文献类型：回顾性队列研究或者前瞻性随机对照研究；（4）观察指标：预后指标包括总生存期（overall survival, OS）和无病生存期（disease-free survival, DFS），合并效应值为风险比（hazard ratio, HR）及其95%CI；（5）文献质量：纽卡斯尔-渥太华量表（Newcastle-Ottawa Scale, NOS）评分≥6分。

两名作者分别进行文献筛选、文献评估及资料提取等工作，通过讨论解决分歧。

### 1.3 统计分析

对于符合纳入标准的每一篇文献均按照作者、发表年份、国家、样本量、检测方法、抗体来源、分析方法和结局变量进行整理（表1^[14,21⇓⇓-24]^）。使用STATA 17.0 （STATA Corp., College Station, TX, USA）进行荟萃分析。OS和DFS采用HR作为合并统计量，并用95%CI表示。使用Chi^2^和I^2^值来评估数据的异质性。如果P<0.05或I^2^>50%，则采用随机效应模型进行分析，如果P≥0.05且I^2^≤50%，则采用固定效应模型进行分析。此外，漏斗图将用于识别发表偏倚，图形分布不对称则表明可能存在发表偏倚，另外使用Egger’s test定量分析发表偏倚，若P<0.05表明发表偏倚有统计学意义。上述统计分析均采用STATA 17.0软件，P<0.05为有统计学差异。

**表1:** 纳入meta分析的5项独立研究的基本特征

Author	Year	Country	Sample size (n)	Detection	Antibody	Cutoff value	Analysis	Outcomes
Bao P, et al.^[14]^	2017	Japan	186	IHC	A mouse monoclonal anti-STMN1 antibody	Mean score	Multivariate analysis	OS/PFS
Nie W, et al.^[21]^	2015	China	113	IHC (Allred score system)	A rabbit anti human STMN1 polyclonal antibody	IHC score>3	Multivariate analysis	DFS
Wang M, et al.^[22]^	2017	China	70	IHC	STMN1 antibody, CST, #13655	Mean score	Multivariate analysis	OS
Shimizu K, et al.^[23]^	2017	Japan	303	IHC	A mouse monoclonal anti-STMN1 (OP18) antibody	IHC score≥3	Multivariate analysis	OS/DFS
Rong B, et al.^[24]^	2017	China	82	IHC	A rabbit anti-stathmin polyclonal antibody	IHC score>2	Multivariate analysis	OS

IHC: immunohistochemistry; DFS: disease-free survival; OS: overall survival; PFS: progression-free survival.

## 2 结果

根据检索条件，共有5篇文献符合纳入标准（表1^[14,21⇓⇓-24]^），文献涉及的754例NSCLC患者被纳入meta分析。5篇研究均采用免疫组化（immunohistochemistry, IHC）方法检测NSCLC细胞和组织中STMN1蛋白的表达，抗体采用鼠源、兔源STMN1单克隆或多克隆抗体，均采用多因素回归分析方法分析STMN1表达对NSCLC患者的预后影响，结局包括OS和DFS。OS纳入文献有4篇，DFS纳入文献有2篇，两者作合并分析。

### 2.1 STMN1表达对NSCLC患者OS的影响

对于STMN1表达与NSCLC患者OS的关系，4篇文献进行了多因素回归分析，meta分析显示，纳入研究之间异质性检验结果提示文献数据之间异质性较小（P=0.668, I^2^=0%），采用固定效应模型进行荟萃分析。结果提示，合并HR为2.28（95%CI: 1.79-2.91, P<0.001），提示治疗后STMN1高表达的OS风险高于STMN1低表达组，说明STMN1表达是NSCLC患者OS的预后影响因素，且STMN1高表达是OS的危险因素（图2）。

**图2:**
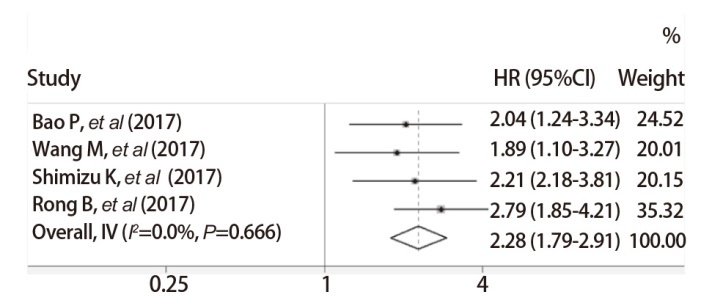
STMN1表达对NSCLC患者OS预后影响的meta分析

### 2.2 STMN1表达对NSCLC患者DFS的影响

对于STMN1表达与NSCLC患者DFS的关系，2篇文献进行了多因素回归分析，meta分析显示，纳入研究之间异质性检验结果提示文献数据之间异质性较小（P=0.791, I^2^=0%），采用固定效应模型进行荟萃分析。结果提示，合并HR为2.14（95%CI: 1.45-3.17, P<0.001），提示治疗后STMN1高表达的DFS风险高于STMN1低表达组，说明STMN1表达是NSCLC患者DFS的预后影响因素，且STMN1高表达是DFS的危险因素（图3）。

**图3:**
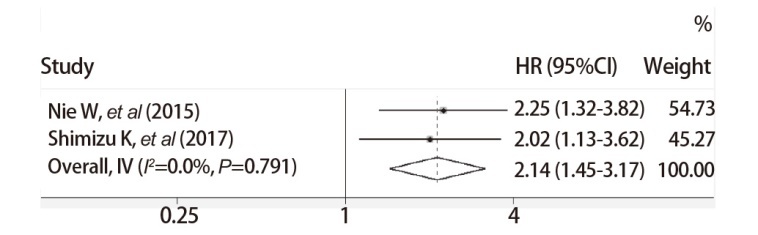
STMN1表达对NSCLC患者DFS预后影响的meta分析

### 2.3 发表偏倚检验

使用HR值的自然对数及其标准误创建漏斗图检测发表偏倚，STMN1表达对NSCLC患者OS预后影响的漏斗图表现出较好的对称性，说明本meta分析不存在发表偏倚（图4）。此外，我们通过Egger’s test定量检测发表偏倚大小，结果提示纳入文献不存在明显发表偏倚（t=-2.78, P=0.109）。

**图4:**
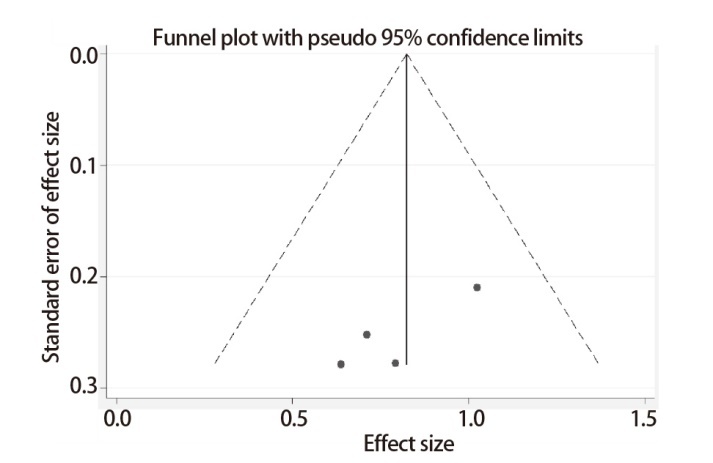
STMN1表达对NSCLC患者OS预后影响的漏斗分析图

## 3 讨论

STMN1是一种微管不稳定蛋白，其去磷酸化形式可以通过促进微管解聚来降低微管稳定性，而微管动力学被认为与肿瘤转移密切相关。近年来研究^[25⇓⇓-28]^表明，STMN1是部分实体瘤预后不良、局部侵袭、淋巴转移、化疗耐药的独立危险因素。与这些研究结论^[25⇓⇓-28]^一致，STMN1的泛分析^[29]^显示，STMN1在大多数癌症类型中均有差异表达。有文献^[30]^报道，在癌症的发展过程中可能存在STMN1-微管动力学-EMT轴。Zeng等^[31]^通过体内和体外实验证实STMN1可通过微管依赖和非微管依赖机制促进NSCLC转移，为STMN1作为抑制转移的治疗靶点提供了理论依据。上述结果提示STMN1是一种致癌基因，可促进NSCLC转移。然而，有研究^[20]^表明STMN1过表达会抑制细胞迁移。

通过meta分析，本文证实STMN1过表达是NSCLC患者预后不良的危险因素，其证据级别和可信度较高。研究人群来自亚洲多个地区，有较好的代表性，对于预测亚洲NSCLC患者的临床预后具有重要的指导意义和更直接、更充实的循证医学证据，结论具有较高普遍适用性。其次，纳入NOS评分≥6分的研究，保证了meta分析的质量。并且除了主要终点OS，本研究将STMN1表达与NSCLC患者DFS之间的相关性进行分析，结果显示，STMN1过表达将导致NSCLC患者DFS获益下降，从OS和DFS两个方面充分证实了STMN1在NSCLC患者预后评估方面具有较高的临床价值。

此外，有两项荟萃分析^[32,33]^研究旨在评估STMN1在恶性肿瘤中的作用。2016年的一项荟萃分析^[32]^揭示了多种癌症中STMN1的表达模式，并阐明了STMN1与癌症之间的关联。该研究纳入了23项研究共3571例患者，结果发现癌症患者的STMN1表达水平高于非癌症患者（OR=0.31），且高STMN1与肿瘤细胞分化（OR=0.73）、淋巴结转移（OR=0.80）及不良临床分期（OR=0.67）相关。最终，该研究得出结论：STMN1高表达的癌症患者OS较短[（27.93±11.54）个月]，而STMN1中低表达的患者OS为（44.81±15.82）个月。另一项荟萃分析^[33]^评估了STMN1在实体癌患者中的预后价值。该研究纳入26项研究共5335例患者，结果表明高STMN1导致OS（HR=2.17）和DFS（HR=2.46）较差^[33]^。这些发现与本研究结果相符。两项研究^[32,33]^均表明STMN1的表达与癌症患者的生存呈负相关。

本文存在部分局限性：（1）纳入的研究均为回顾性研究，未纳入更高质量的前瞻性随机对照研究；（2）所有纳入的研究中，选择偏差是不可避免的；（3）针对DFS的meta分析所纳入的研究较少，存在异质性；（4）各研究中关于STMN1表达的cut-off值的定义标准不统一，可能对结果存在一定程度的影响。

综上所述，本文通过纳入5项研究，包括754例研究对象，证实STMN1是NSCLC患者预后不良的一个危险因素，STMN1过表达的NSCLC患者OS、DFS更差。但上述发现还需更多的相关研究进一步予以论证。
